# The complete chloroplast genome sequence of *Michelia floribunda*

**DOI:** 10.1080/23802359.2020.1730730

**Published:** 2020-02-28

**Authors:** Yongkang Sima, Yunqing Li, Yi Wang

**Affiliations:** Laboratory of Forest Plant Cultivation and Utilization, Yunnan Academy of Forestry and The Key Laboratory of Rare and Endangered Forest Plants of State Forestry Administration, Kunming, Yunnan, People’s Republic of China

**Keywords:** *Michelia floribunda*, chloroplast, Illumina sequencing, phylogenetic analysis

## Abstract

The first complete chloroplast genome (cpDNA) sequence of *Michelia floribunda* was determined from Illumina HiSeq pair-end sequencing data in this study. The cpDNA is 160,049 bp in length, contains a large single-copy region (LSC) of 88,140 bp and a small single-copy region (SSC) of 18,773 bp, which were separated by a pair of inverted repeats (IR) regions of 26,568 bp. The genome contains 132 genes, including 87 protein-coding genes, 8 ribosomal RNA genes, and 37 transfer RNA genes. Further phylogenomic analysis showed that *M. floribunda* was closely related to *Michelia yunnanensis* in Michelieae tribe.

*Michelia floribunda* is the species of the genus *Michelia* within the family Magnoliaceae, native in Yunnan, Sichuan, Hubei of China and Myanmar. It is an excellent timber and ornamental plant (Zhu et al. 2012). The leaves of *M. floribunda* have rich content of sesquiterpene lactones (Xiong et al. 2001). The extract of *M. floribunda* also showed antibacterial and anticancer activities (Zhao 2005). The pyrethroid from *M. floribunda* has insecticidal activity (Mondranondra et al. 1990). However, there has been no genomic studies on *M. floribunda*.

Herein, we reported and characterized the complete *M. floribunda* plastid genome. The GenBank accession number is MN897728. One *M. floribunda* individual was collected from Kunming arboretum, Yunnan Academy of Forestry, Yunnan Province of China (25°14′48ʺN, 102°75′53ʺE). The specimen is stored at Yunnan Academy of Forestry Herbarium, Kunming, China, and the accession number is S97156. DNA was extracted from its fresh leaves using DNA Plantzol Reagent (Invitrogen, Carlsbad, CA, USA).

Paired-end reads were sequenced using Illumina HiSeq system (Illumina, San Diego, CA). In total, about 8.8 million high-quality clean reads were generated with adaptors trimmed. Aligning, assembly, and annotation were conducted by CLC *de novo* assembler (CLC Bio, Aarhus, Denmark), BLAST, GeSeq (Tillich et al. 2017), and GENEIOUS v 11.0.5 (Biomatters Ltd, Auckland, New Zealand). To confirm the phylogenetic position of *M. floribunda*, other 10 species of *Michelieae* tribe from NCBI were aligned using MAFFT v.7 (Katoh and Standley 2013). The auto-algorithm in the MAFFT alignment software was used to align the 13 complete genome sequences and the G-INS-i algorithm was used to align the partial complex sequences. The maximum likelihood (ML) bootstrap analysis was conducted using RAxML (Stamatakis 2006); bootstrap probability values were calculated from 1000 replicates. *Liriodendron tulipifera* (MK477550) and *Liriodendron chinense* (KU170538) were served as the out-group.

The complete *M. floribunda* plastid genome is a circular DNA molecule with the length of 160,049 bp, contains a large single-copy region (LSC) of 88,140 bp and a small single-copy region (SSC) of 18,773 bp, which were separated by a pair of inverted repeats (IR) regions of 26,568 bp. The overall GC content of the whole genome is 39.2%, and the corresponding values of the LSC, SSC, and IR regions are 37.9%, 34.3%, and 43.2%, respectively. The plastid genome contained 132 genes, including 87 protein-coding genes, 8 ribosomal RNA genes, and 37 transfer RNA genes. Phylogenetic analysis showed that *M. floribunda* was closely related to *Michelia yunnanensis* in Michelieae tribe (Figure 1). The determination of the complete plastid genome sequences provided new molecular data to illuminate the Michelieae tribe in Magnoliaceae family evolution.

**Figure 1.**
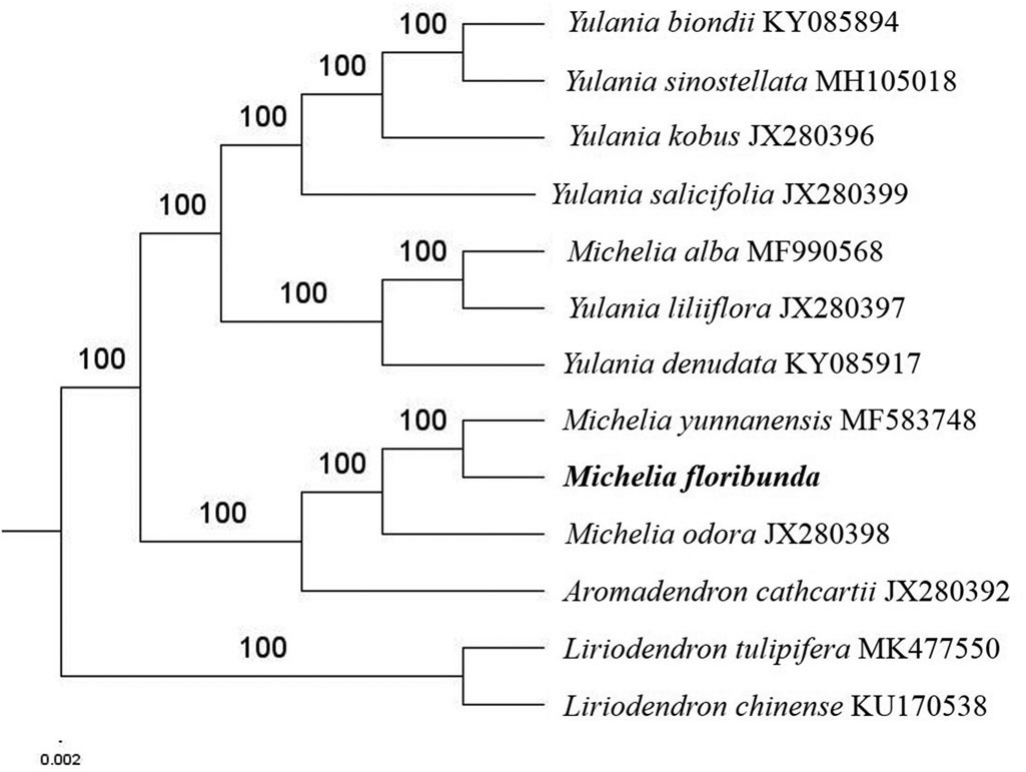
The maximum-likelihood tree based on the eleven chloroplast genomes of Michelieae tribe in Magnoliaceae family. The bootstrap value based on 1000 replicates is shown on each node.
